# Phenological and intrinsic predictors of mite and haemacoccidian infection dynamics in a Mediterranean community of lizards

**DOI:** 10.1017/S0031182021000858

**Published:** 2021-09

**Authors:** Robby M. Drechsler, Josabel Belliure, Rodrigo Megía-Palma

**Affiliations:** 1 Cavanilles Institute of Biodiversity and Evolutionary Biology, University of Valencia, c/ Catedrático José Beltrán Martínez 2, E-46980 Paterna, Valencia, Spain; 2 Department of Life Sciences, Global Change Ecology and Evolution Group (GLOCEE), Universidad de Alcalá (UAH), E-28805, Alcalá de Henares, Madrid, Spain; 3 Department of Biomedicine and Biotechnology, Universidad de Alcalá (UAH), Parasitology Area, Campus Universitario, E-28805, Alcalá de Henares, Madrid, Spain; 4 CIBIO-InBIO: Research Center in Biodiversity and Genetic Resources, University of Porto, P-4485-661, Vairão, Portugal

**Keywords:** Ecological interactions, host–parasite dynamics, Iberian Peninsula, Lacertidae, parasite phenology

## Abstract

Ectotherms are vulnerable to environmental changes and their parasites are biological health indicators. Thus, parasite load in ectotherms is expected to show a marked phenology. This study investigates temporal host–parasite dynamics in a lizard community in Eastern Spain during an entire annual activity period. The hosts investigated were *Acanthodactylus erythrurus*, *Psammodromus algirus* and *Psammodromus edwardsianus*, three lizard species coexisting in a mixed habitat of forests and dunes, providing a range of body sizes, ecological requirements and life history traits. Habitat and climate were considered as potential environmental predictors of parasite abundance, while size, body condition and sex as intrinsic predictors. Linear models based on robust estimates were fitted to analyse parasite abundance and prevalence. Ectoparasitic mites and blood parasites from two haemococcidian genera were found: *Lankesterella* spp. and *Schellackia* spp. Habitat type was the only predictor explaining the abundance of all parasites, being mostly higher in the forest than in the dunes. The results suggest that particularities in each host–parasite relationship should be accounted even when parasites infect close-related hosts under the same environmental pressures. They also support that lizard parasites can be biomarkers of environmental perturbation, but the relationships need to be carefully interpreted for each host–parasite assemblage.

## Graphical abstract

**Figure:**
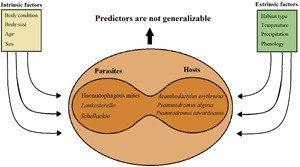

## Introduction

Parasites thrive to the expense of other organisms and are usually part of intricate ecological webs. The presence of high parasite diversity is considered a reliable indicator of good environmental quality because of the ecological equilibrium presumed for host–parasite relationships (Marcogliese, 2005). Consequently, understanding the dynamics of host–parasite interactions has been a major aim in evolutionary ecology, and studies at the community scale are needed if we want to understand the influence that hosts and parasites diversity have on each other (Vázquez *et al*., 2005, 2007).

The Western Mediterranean is a biodiversity hot spot of both hosts and parasites, and reptiles constitute a good model of the complexity involved in such interactions (Molina-Venegas *et al*., 2015; Megía-Palma *et al*., 2018*a*). Multiple factors influence the outcome of the interaction, one is stress, and sources of stress for hosts can be either environmental or intrinsic. For example, individuals subjected to stressful environments can reallocate energy to body functions other than immune defence to cope with stress (Adamo *et al*., 2017). This may increase their susceptibility to parasitic infections (Oppliger *et al*., 1998). Thus, variation in parasite abundance in correlation with environmental gradients of stress can be interpreted as biomarkers of environmental costs on the hosts’ immune defences (Megía-Palma *et al*., 2020*a*). Besides spatial covariation with environmental stress, the abundance of parasites may vary locally with phenology (e.g. McDevitt-Galles *et al*., 2020). However, temporal dynamics of parasite abundance have scarcely been studied in particular hosts such as reptiles (Schall and Marghoob, 1995).

In the last two decades, parasitologists have been unveiling the diversity of parasites infecting Mediterranean lizards (Galdón *et al*., 2006; Maia *et al*., 2011, 2012; Megía-Palma *et al*., 2014, 2018*a*). Blood parasites that infect lizards of this region cluster in two distinct phylogenetic groups. The most common are haemogregarines (Roca and Galdón, 2010; Maia *et al*., 2014; Álvarez-Ruiz *et al*., 2018; Megía-Palma *et al*., 2020*b*). They are transmitted by haematophagous mites of the genus *Ophionyssus* (Reichenow, 1919; Svahn, 1975; Haklová-Kočíková *et al*., 2014), and the potential drivers governing their prevalence and intensity are only starting to be understood (Álvarez-Ruiz *et al*., 2018; Megía-Palma *et al*., 2020*a*).

A second and less common group of blood parasites of lizards in the Mediterranean region are haemococcidians, which are highly host-specific (Megía-Palma *et al*., 2018*a*). However, the factors governing their prevalence and distribution are even less understood compared to haemogregarines. The haemococcidian genera *Schellackia* spp. and *Lankesterella* spp. (order Eimeriida) infect Iberian lizards (Maia *et al*., 2014; Megía-Palma *et al*., 2014, 2018*a*). Parasites of the former genus undergo several replication cycles of sexual and asexual reproduction in lizard hosts, whereas in the gut of haematophagous mites the parasite only becomes dormant (Telford, 2009). The cycle of *Lankesterella* spp. in the Mediterranean is unknown, but dipteran and acarine arthropods are competent vectors of American lankesterellids (Megía-Palma *et al*., 2017). Hematic stages (i.e. sporozoites) of *Schellackia* spp. and *Lankesterella* spp. in Iberian lizards are morphologically distinguished by the differential number of refractile structures in the cytoplasm; *Schellackia* spp. shows one refractile body, while sporozoites of *Lankesterella* spp. show two (Megía-Palma *et al*., 2014, 2018*a*).

Factors explaining parasite abundances are multiple, and intrinsic and extrinsic predictors, as well as particularities in the life history traits of both hosts and parasites, may interact to shape host–parasite dynamics (Klukowski, 2004; Illera *et al*., 2017; Padilla *et al*., 2017). For example, previous studies found positive relationships between body size and the abundance of haemogregarine infection in small to medium-sized lizards (Amo *et al*., 2005; Molnár *et al*., 2013; Maia *et al*., 2014). Those studies used body size as a proxy for age because lizards have indeterminate growth and infection likelihood might increase with age, as older individuals accumulate exposure to parasites over time (e.g. Schall and Marghoob, 1995). Sex is usually an important intrinsic factor associated with increased susceptibility to infections (Folstad and Karter, 1992; Saino *et al*., 1995; Alonso-Alvarez *et al*., 2007). In lizards, although the specific effect of sex on blood parasites remains unclear, there is consensus that sexual hormones increase the susceptibility to ectoparasites (reviewed in Roberts *et al*., 2004 but also see Veiga *et al*., 1998). Furthermore, host–parasite dynamics may vary along the lizards’ period of activity, as both environmental abundance of parasites and hosts’ susceptibility to infections may show phenological variation (Klukowski, 2004; Huyghe *et al*., 2010; Megía-Palma *et al*., 2020*c*).

As commented, sources of stress are positively associated with haemogregarine abundances in lizards (Oppliger *et al*., 1996, 1998; Megía-Palma *et al*., 2020*a*). One important source of environmental stress in Mediterranean habitats may be an increasing constriction in the availability of favourable thermal microhabitats for lizards due to raising temperatures towards summer (Díaz *et al*., 2006; Vickers *et al*., 2011). Although lizards in Mediterranean environments may acclimate to increasing temperatures by accommodation of their thermal preferences (Díaz *et al*., 2006; Megía-Palma *et al*., 2020*c*), this thermo-physiological shift might have costs on lizards (Vickers *et al*., 2011). Thermal restrictions have immunosuppressant effects on lizards (Han *et al*., 2020), with gravid females demonstrating higher thermal sensitivity influenced by an additive effect of dehydration (Dupoué *et al*., 2020; Megía-Palma *et al*., 2020*a*). Stressed lizards, by this or other reasons, exhibit a lower ability to heal cutaneous wounds or a reduced immune response (Lucas and French, 2012; Sprayberry *et al*., 2019; Han *et al*., 2020). Thus, parasitic transmission and/or replication of some parasites may be facilitated in immunosuppressed lizards (e.g. Salvador *et al*., 1996; Megía-Palma *et al*., 2020*a*). Intra or interspecific (social) interactions may also be an important source of environmental stress, with potential influence on lizards’ susceptibility to infections (Mugabo *et al*., 2015). Indeed, Oppliger *et al*. (1998) experimentally demonstrated that the increase in the intensities of haematic parasites in *Zootoca (=Lacerta) vivipara* (Lichtenstein, 1823) reflected stress, being higher in environments with higher predation pressure and intraspecific competition associated with increased release of glucocorticoids. Similarly, Carbayo *et al*. (2019) found that the Algerian sand racer, *Psammodromus algirus*, has more blood parasites in poor quality habitats where lizards also had worse body condition.

The aim of this study was to investigate the phenological host–parasite dynamics in a community of Mediterranean lizards during a one-year period of lizard activity (May–October; the rest of the year they hibernate and remain inactive in burrows). The selected hosts were three co-habiting lizard species that provide a range of sizes, ecological requirements and life history traits: *Acanthodactylus erythrurus* (Schinz, 1834), *P. algirus* (Linnaeus, 1758) and *Psammodromus edwardsianus* (*P. hispanicus*) (Dugès, 1829). The three species differ in their habitat preference, with *A. erythrurus* preferring more open habitats with sandy substrate, while both *Psammodromus* species prefer higher vegetation cover provided by forests with dense undergrowths and leaf litter (Escarré and Vericad, 1981; Arnold, 1987; Díaz and Carrascal, 1991). Despite all three lizard species being insectivorous, they also present certain dietary differences: *A. erythrurus* is known to show a marked preference for ants (Pollo and Pérez-Mellado, 1991), *P. algirus* shows the greater variety of insects in the diet (Salvador, 2015), and *P. edwardsianus* consumes mainly small and hard prey like Coleoptera or Hemiptera (Fitze, 2012). *Acanthodactylus erythrurus* is the biggest [up to 8 cm snout-vent length (SVL)] and most thermophilic species of the three (Belliure *et al*., 1996; Verwaijen and Van Damme, 2007). Although little is known about the exact life span of the species in the wild, the results of several studies allow to order the species from shorter to longer expected life span (Carretero and Llórente, 1991; Drechsler and Monrós, 2019; Comas *et al*., 2020). Following this statement, *A. erythurus* has an intermediate life span, which is not known in detail, but the results in Drechsler and Monrós (2019), with 80% yearly renewal indicate that it seems to be around two years for most individuals in this population. For *P. algirus*, some studies describe interannual survival rates of 25% for juveniles and 35% for adults (Salvador, 2015), indicating that the species has the longest life span, as an important part of individuals survive more than 2 years. Finally, *P. edwardsianus* is the smallest (maximum SVL of 5–6 cm) and has the shortest life span (with a near 100% renewal of the population every year; Fitze, 2012).

Significant predictors of the prevalence and abundances of haematophagous mites and blood parasites were analysed in the three lizard species at two contrasting habitats where they coexist. Based on differences in their life history strategies, we predict that environmental variables will affect differently host–parasite dynamics in the three lizard species. We also predict that females of the less thermophilic species, *P. edwarsianus* and *P. algirus*, will have a higher parasite load during the warmer period, which coincides with the critical period of clutch development (Carretero, 2006; Dupoué *et al*., 2020). Furthermore, we will also test whether lizard species of intermediate longevity will have intermediate abundances of infection. The latter hypothesis will be supported if the abundance of parasites in *P. edwardsianus* < *A. erythrurus* < *P. algirus*, according to differences in their life span expectancy.

## Material and methods

### Study area

The study area is situated in East Spain, about 10 km South from Valencia City and is part of the Albufera de Valencia Natural Park (39°20′20″N 0°18′43″W). It is a coastal line of sandy substrate about 10 km long (N-S) and 1 km wide (E-W) in the Western coast of the Mediterranean Sea, with a gradient of vegetation cover increasing from the coastal sand dune area to the inland pine forest (Ibor and Matarredona, 2016).

Lizards were captured in both dune and forest habitats. The ‘dunes’ are characterized by bare sand sparsely covered by herbaceous and bush species of plants, providing low heterogeneity of thermal microhabitats. The preponderant height of plants in this habitat is less than 1 m (*Ammophila arenaria*, *Helichrysum stoechas*, *Euphorbia paralias*, *Medicago marina* and *Rhamnus alaternus*, among others). The ‘forest’ substrates are fixed sand dunes dominated by Aleppo pines (*Pinus halepensis*) and a dense undergrowth vegetation (*Smilax aspera*, *Asparagus officinalis*, *Chamaerops humilis* and *Pistacia lentiscus*, among others) that provide more heterogeneity of thermal microhabitats.

### Fieldwork

Field sampling was carried out in 2017 between May and October, to cover a one-year period of activity of the three species of lizards at the area. Lizard counting and capturing was performed by one researcher (RD) twice per week in each habitat type (alternating sampling areas), by randomly walking the area for two hours beginning about three hours after sunrise. Random walks were used instead of fixed transects because (i) this allowed to avoid the repeated handling effect and double counting, changing the trajectory each day; and (ii) it was more suitable, especially in the forest habitat, given the dense undergrowth vegetation which often blocked the way. Days with suboptimal meteorological conditions for lizard activity (rain, strong wind, important cloud coverage, etc.) were not sampled. Lizards were captured by hand or noosing (e.g. Guillén-Salazar *et al*., 2007), and put in individual cloth bags until processing. All individuals were measured (SVL) using a ruler, to the nearest 1 mm, and weighed using a digital scale, with a precision of 0.01 g, and species and sex were identified. In the case of females, the gravidity status was assessed by palpation. Finally, the number of visible ectoparasites on each individual was counted, blood samples were obtained from toe-clipping and smeared on a microscope slide. Resampling the lizards was avoided by assigning them a unique code by toe-clipping (Bellairs and Bryant, 1968; Perry *et al*., 2011; Barrientos and Megía-Palma, 2021). The lizards were released near the corresponding capture point.

### Blood sample processing

Blood smears were fixed with 100% methanol and stained them with 1:10 solution of Giemsa for 40 min. The blood smears were screened at ×1000 magnification in a light microscope (CX41, Olympus, Tokyo, Japan). Blood parasites were counted in 5000 blood cells (Megía-Palma *et al*., 2016). The genera of parasites found were identified by morphological characters (Megía-Palma *et al*., 2014, 2018*a*; Álvarez-Ruiz *et al*., 2018). Particularly, the presence and the number of refractile bodies were key diagnostic characteristics (i.e. Megía-Palma *et al*., 2014) (Fig. S1).

### Data analysis

The daily abundance of lizards was standardized dividing the number of observed lizards of each species per day by the duration of the census (obtaining values in individuals/hour). For the statistical analysis, recaptures and individuals with incomplete datasets were not included. The differences in lizard abundance were analysed fitting a Generalized Linear Model (GLM) with *γ* error distribution with a log linking function, considering species, habitat, month and the triple interaction species × habitat × month as predictor variables, computing the results by a type II ANOVA.

Given the sexually asynchronous cycles in fat body development in Mediterranean lizards (Carretero, 2006), a body condition index (i.e. BCI) was calculated as the residuals of the relation between both log_10_-transformed SVL and body mass (e.g. Drechsler *et al*., 2020). This was done separately for each sex and species to remove confounding effects (Álvarez-Ruiz *et al*., 2018; Megía-Palma *et al*., 2020*a*). The differences in body condition between habitat types were analysed performing an ANOVA for each species separately, considering the interaction with sex. In the case of *A. erythrurus*, the age of each individual was also estimated, following Drechsler and Monrós (2019). In brief, the regression formula of the growth curve was applied for each sex and a delay in growth due to hibernation was considered, when necessary.

Abundance of each parasite species was analysed considering the number of parasites per host (Rózsa *et al*., 2000). Given the high proportion of uninfected lizards in the sample, fitting a model to the data that fulfilled the parametric assumptions of residual normality and homoscedasticity was not possible. Nor negative binomial, zero-inflated models or log_10_-transformation of the data improved the residual distribution of the model. Thus, a robust estimate GLM (GLM-rob) (Cantoni and Ronchetti, 2001) with Poisson error distribution linked to a log linking function was implemented, through the ‘glmrob’ function of the ‘robustbase’ package. The prevalence of each parasite species was analysed by fitting the percentage of infested individuals to a GLM with binomial error distribution and logit link function, the results were computed by a type II ANOVA.

The following predictors for parasite abundance and prevalence were considered: host species, habitat type, climate, month, sex, SVL, body condition and interactions of species with habitat, SVL, sex, month and month with sex. Mite abundance was considered in the case of blood parasites, and gravidity was tested in the case of females. Finally, a Spearman correlation test was carried out to test if the infection parameters (prevalence, mean and median intensity) were correlated to climatic variables (monthly mean temperature, mean maximum and minimum temperatures, and accumulated precipitation), which were obtained from a meteorological station situated less than 10 km from the study area (Racó de l'Olla; https://www.avamet.org/mx-mes.php?id=c15m250e27). In this analysis, intensity (considering only infected lizards) was used instead of abundance (i.e. infected and uninfected lizards considered) (*sensu* Rózsa *et al*., 2000) for three reasons: (i) this allowed us to analyse the effect of climatic fluctuations strictly on the host–parasite relationship, as uninfected lizards lack parasites; (ii) the high proportion of uninfected lizards would bias the results; and (iii) this analysis is unifactorial, allowing to use a smaller sample size than the multifactorial approach of previous analyses. *Psammodromus edwardsianus* was excluded in all analysis of blood parasites, as only one lizard was infected. All the statistical analyses were ran using the statistics software R v4.0.3 (R Core Team, 2020).

## Results

The dataset included 256 individuals (157 *A. erythrurus*, 51 *P. algirus* and 48 *P. edwardsianus*). The abundances of all lizard species were significantly higher in the forest (Table S1); a constant effect observed across the activity period (Fig. S2). Body condition did not differ between habitats for the three species (*F*_12,104_ = 1.058, *P* = 0.340; Table 1).

**Table 1.** Mean ± s.e. of lizard body condition (×100) of each species in both considered habitat types, with the corresponding sample size (*n*, in lizards) and the results of the statistical analysis in each case

	Habitat		ANOVA		
Species	Forest	Dunes	d.f.|*n*	*F*	*P*
*A. erythrurus*	0.47 ± 1.09 (*n* = 68)	−0.36 ± 0.82 (*n* = 89)	1 | 157	0.382	0.538
*P. algirus*	0.26 ± 1.97 (*n* = 22)	−0.20 ± 1.81 (*n* = 29)	1 | 51	0.028	0.868
*P. edwardsianus*	0.46 ± 3.12 (*n* = 29)	−0.71 ± 3.02 (*n* = 19)	1 | 48	0.063	0.803

### Parasite prevalence

**Fig. 1.**
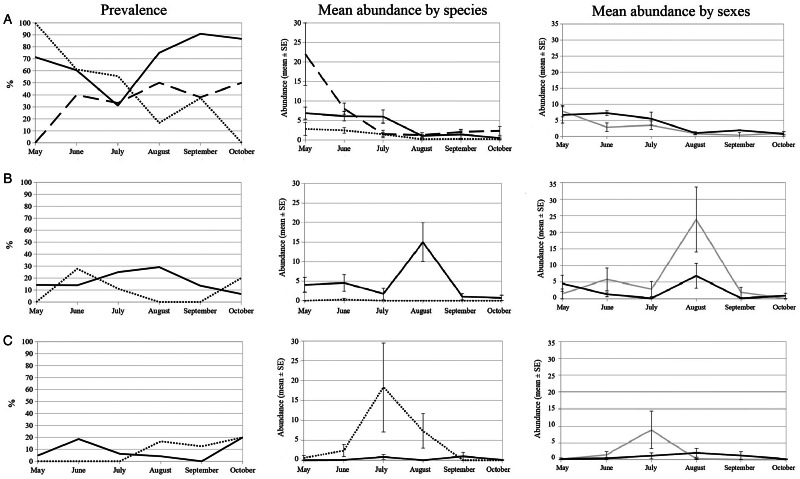
Representation of the seasonal variation of infection parameters of each parasite (A: mites, B: *Lankesterella* spp. and C: *Schellackia* spp.) and each host species: *A. erythrurus* (solid line), *P. algirus* (dotted line) and *P. edwardsianus* (dashed line). From left to right: the prevalence, expressed as a percentage of infected individuals; the comparison between species of mean ± s.e. of infection abundance and the comparison between males (black) and females (gray) of mean ± s.e. infection abundance.

**Fig. 2.**
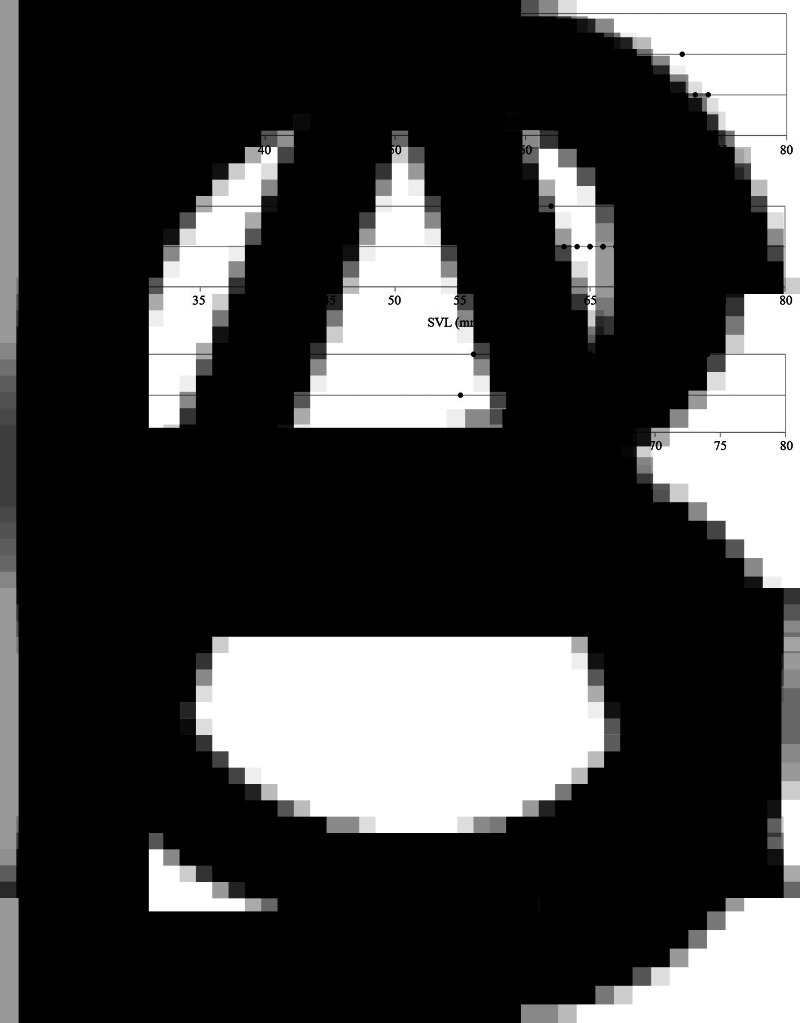
Distribution of infected individuals among the snout-vent length (SVL) range of each host species: mites (A), *Lankesterella* spp. (B), and *Schellackia* spp. (C).

Mites were more prevalent than blood parasites in the three host species (Table 2). Month and sex significantly explained the prevalence of mites (Table 3), while month differently affected the host species (Fig. 1), the effect of sex was consistently higher in males (59.9%) than females (52.5%) through all host species. The relationship between the prevalence of mites and the SVL of the lizards was significantly different among host species (Table 3), being mites present in smaller body sizes in *P. edwardsianus* than in the other two species (Fig. 2).

**Table 2.** Mean ± s.e. infection abundance of each parasite and prevalence (in brackets) for each host species

	*A. erythrurus*	*P. algirus*	*P. edwardsianus*
Mites	4.21 ± 0.58 (59%)	1.47 ± 0.33 (49%)	3.33 ± 0.77 (58%)
*Lankesterella* spp.	4.66 ± 1.09 (22%)	0.12 ± 0.09 (4%)	0.00 (0%)
*Schellackia* spp.	0.34 ± 0.19 (4%)	5.02 ± 2.21 (24%)	0.02 ± 0.02 (2%)

**Table 3.** Results of the general linear models for the parasite prevalence of the different parasites: residual deviance (Dev), residual degrees of freedom (d.f.) and *F* and *P* statistics

	Mites				*Lankesterella* spp.				*Schellackia* spp.			
Factor	Dev	d.f.	*F*	*P*	Dev	d.f.	*F*	*P*	Dev	d.f.	*F*	*P*
Species	0.207	254	0.207	0.650	**11.278**	**206**	**11.278**	**<0.001**	**14.268**	**206**	**14.268**	**<0.001**
Habitat	2.624	253	2.624	0.105	2.196	205	2.196	0.138	0.817	205	0.817	0.366
Month	**40.735**	**252**	**40.736**	**<0.001**	0.222	204	0.223	0.637	0.011	204	0.011	0.916
Sex	**5.674**	**251**	**5.674**	**0.017**	2.347	203	2.347	0.126	0.688	203	0.688	0.407
SVL	0.282	250	0.282	0.595	**25.598**	**202**	**25.958**	**<0.001**	2.869	202	2.870	0.090
BCI	0.272	249	0.272	0.602	1.878	201	1.878	0.171	0.529	201	0.529	0.467
Mite abundance	–	–	–	–	0.168	200	0.168	0.682	1.370	200	1.371	0.242
Species × habitat	2.559	248	2.555	0.110	**3.940**	**199**	**3.940**	**0.047**	**7.103**	**199**	**7.103**	**0.008**
Species × SVL	**4.673**	**247**	**4.673**	**0.031**	3.034	198	3.035	0.081	0.010	198	0.010	0.919
Species × sex	0.116	246	0.116	0.733	1.715	197	1.713	0.191	0.007	197	0.007	0.933
Month × sex	1.077	245	1.077	0.299	3.033	196	3.033	0.082	0.324	196	0.324	0.569
Species × month	0.035	244	0.035	0.851	**4.145**	**195**	**4.146**	**0.042**	2.767	195	2.767	0.096

Significant results (*P* < 0.05) are shown in bold.

None haemogregarine blood parasites were found in the study, but two genera of haemococcidians: *Lankesterella* spp. and *Schellackia* spp. (Fig. S1). Blood parasites of the genus *Lankesterella* spp. were found almost exclusively infecting *A. erythrurus* (35 out of the 37 lizards infected; Table 2). Significant effects were detected of species and SVL (the correlation was positive and consistent through species) on the prevalence of *Lankesterella* spp. (Table 3, Fig. 2). The interactions of species with habitat type and species with month were also significant (Table 3). The prevalence of *Lankesterella* spp. in *A. erythrurus* was higher in the forest (29.4%) than in the dunes (16.9%). None *P. algirus* was infected by *Lankesterella* spp. in the forest and the prevalence in the dunes was 6.9%. The maximum prevalence of *Lankesterella* spp. in *A. erythrurus* was in August. However, no *P. algirus* was found infected in this month (Fig. 1).

**Fig. 3.**
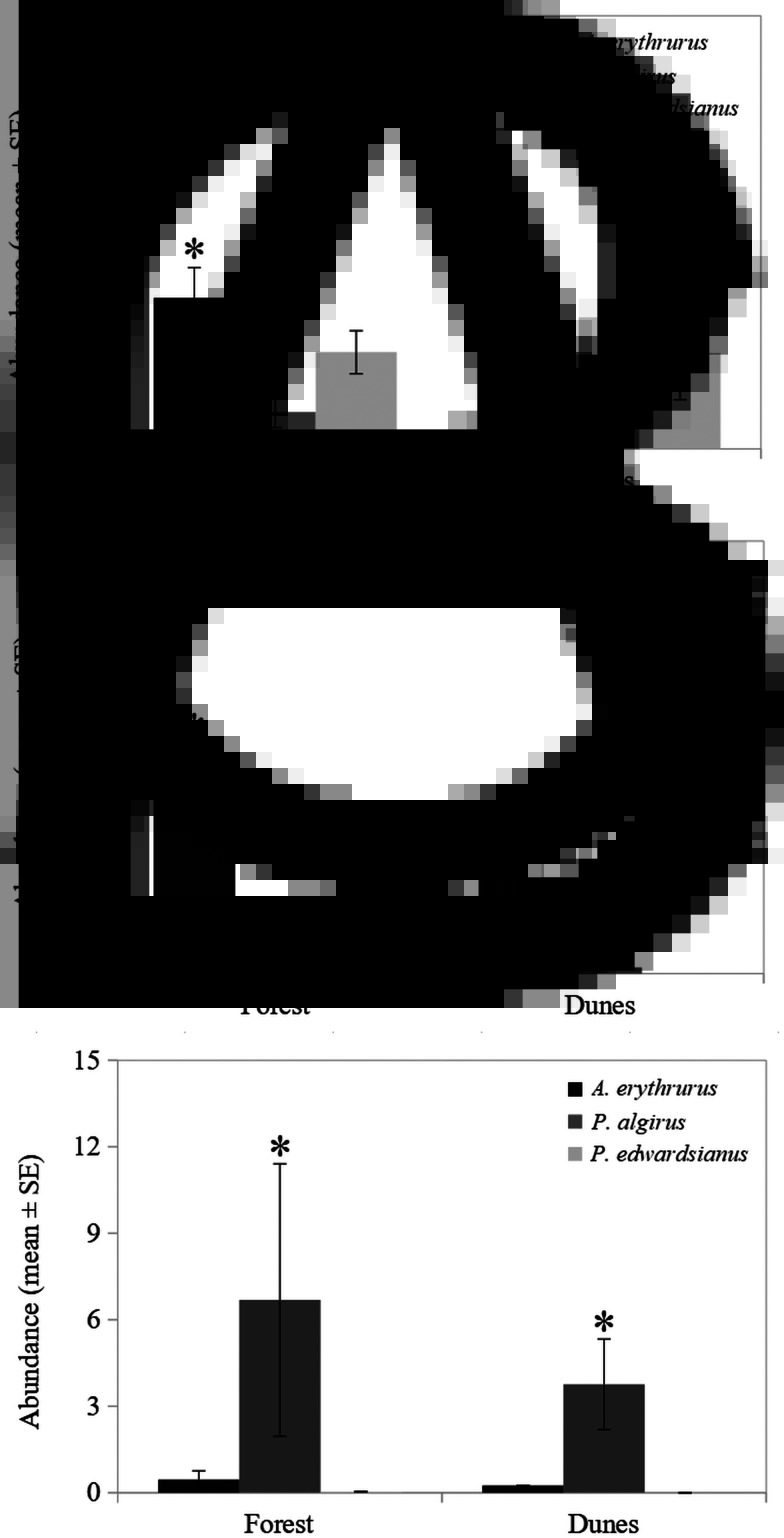
Mean ± SE of the abundance of mites (A), *Lankesterella* spp. (B) and *Schellackia* spp. (C) in both habitats for each host species: *A. erythrurus* (black), *P. algirus* (dark grey) and *P. edwardsianus* (light grey). Significant differences between habitats are indicated by asteriscs.

*Schellackia* spp. was the main blood parasite found in *P. algirus* (12 out of the 20 lizards infected), but it also infected the other two lizard species (7 out of 20 *A. erythrurus*; 1 out of 20 *P. edwardsianus*; Table 2). The interaction between species and habitat significantly explained prevalence (Table 3). The same pattern as in the case of *Lankesterella* spp. could be observed; the prevalence of *Schellackia* spp. in *A. erythrurus* was higher in the forest habitat (8.8%) than in the dunes (1.1%), while in *P. algirus*, the prevalence of this parasite was higher in the dunes (27.6%) than in the forest (18.2%).

Mites infested all ranges of body sizes of the three lizard species. Blood parasites were only found in individuals older than ~300 days in the case of *A. erythrurus* (Table S2), while they did infect a very small (i.e. young, SVL = 39 mm) individual of *P. algirus*. In the case of *P. edwardsianus*, only one individual was infected by blood parasites.

### Parasite abundance

**Fig. 4.**
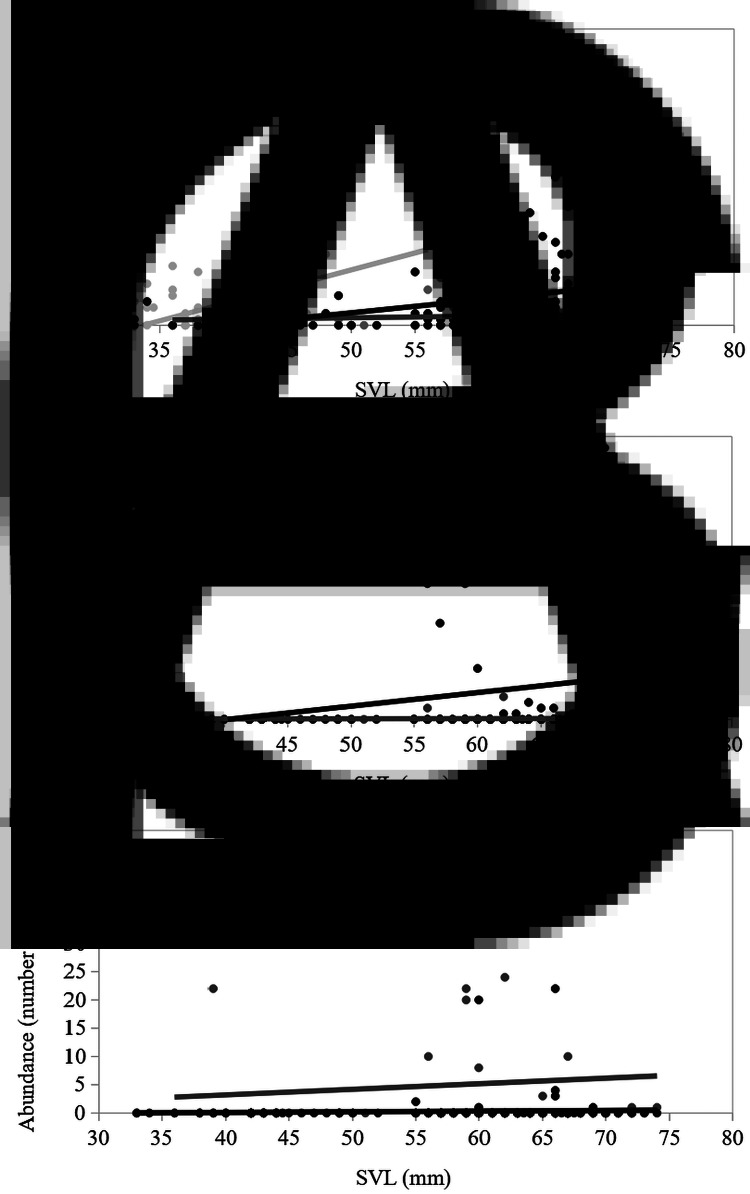
Correlation of parasite abundance (A: mites, B: *Lankesterella* spp. and C: *Schellackia* spp.) with snout-vent length (SVL) for each host species: *A. erythrurus* (black), *P. algirus* (dark grey) and *P. edwardsianus* (light grey). Line of best fit included to show relationship.

Nearly all predictors significantly explained mite abundance (Table 4). In *A. erythrurus*, mites were more abundant in the forest than in the dunes; in *P. algirus*, it was the opposite; and in *P. edwardsinaus*, the mite abundances were similar between habitats (Fig. 4). Males of *A. erythruru*s had higher infestation rates by mites [4.9 ± 0.71 (s.e.)] than females [2.94 ± 0.97], but females were more intensely infested in both species of the genus *Psammodromus*: males 1.30 ± 0.46 (in *P. algirus*) and 2.39 ± 0.53 (in *P. edwardsianus*) and females 1.57 ± 0.47 (in *P. algirus*) and 5.40 ± 2.13 (in *P. edwardsianus*). In all host species, mites were more abundant in spring and early summer, with a higher peak in *P. edwardsianus* (Table 4, Fig. 1). The SVL showed a positive correlation with mite infestation in all species, especially in *P. edwardsianus* (Fig. 4). However, it was only significant for *A. erythrurus* (Spearman correlation test, rho = 0.368, *P* < 0.001 for *A. erythrurus*; rho = 0.011, *P* = 0.938 for *P. algirus*; and rho = 0.252, *P* = 0.085 for *P. edwardsianus*). The phenology of mite infestation did not differ significantly between sexes, males presenting slightly higher mite abundances in June (Table 4, Fig. 1). All the interactions of the predictors with species significantly explained mite abundance (Table 4).

**Table 4.** Robust estimates of parasite abundance for the different parasites: estimate (*E*_st_), standard error (s.e.) and *z* and *P* statistics

	Mites				*Lankesterella* spp.				*Schellackia* spp.			
Factor	*E*_st_	s.e.	*z*	*P*	*E*_st_	s.e.	*z*	*P*	*E*_st_	s.e.	*z*	*P*
Species	**4.166**	**0.484**	**8.596**	**<0.001**	**44.910**	**16.919**	**2.654**	**0.008**	−0.553	5.519	−0.100	0.920
Habitat	**−0.719**	**0.195**	**−3.690**	**<0.001**	**−1.833**	**0.202**	**−9.070**	**<0.001**	**−5.464**	**2.448**	**−2.232**	**0.026**
Month	**−0.682**	**0.252**	**−2.703**	**0.007**	1.974	1.513	1.305	0.192	0.453	0.980	0.463	0.644
Sex	**1.651**	**0.310**	**5.321**	**<0.001**	**−8.152**	**2.489**	**−3.274**	**0.001**	−1.152	2.482	−0.464	0.642
SVL	**0.105**	**0.015**	**6.920**	**<0.001**	**1.322**	**0.235**	**5.605**	**<0.001**	0.080	0.121	0.667	0.505
BCI	0.488	0.450	1.085	0.278	**−6.526**	**0.893**	**−7.301**	**<0.001**	−1.008	2.256	−0.447	0.655
Mite abundance	–	–	–	–	**−0.066**	**0.031**	**−2.104**	**0.035**	−0.048	0.089	−0.543	0.587
Species × habitat	**0.588**	**0.119**	**4.938**	**<0.001**	–	–	–	**–**	**3.393**	**1.361**	**2.492**	**0.013**
Species × SVL	**−0.056**	**0.008**	**−6.675**	**<0.001**	**−0.764**	**0.225**	**−3.391**	**<0.001**	−0.018	0.069	−0.270	0.787
Species × sex	**−0.817**	**0.155**	**−5.260**	**<0.001**	3.703	2.355	1.573	0.116	−0.086	1.184	−0.073	0.942
Month × sex	0.160	0.110	1.454	0.146	0.192	0.131	1.463	0.143	0.234	0.370	0.632	0.527
Species × month	**−0.174**	**0.065**	**−2.674**	**0.007**	−1.894	1.496	−1.267	0.205	−0.458	0.403	−1.136	0.256

Significant results (*P* < 0.05) are shown in bold.

Habitat type, sex, body condition, mite abundance and the interaction of species with SVL significantly explained the abundance of *Lankesterella* spp. (Table 4). *Lankesterella* spp. was more abundant in the forest than in the dunes (Fig. 3). The abundance of *Lankesterella* spp. showed a negative relationship with body condition (Table 4). Similarly, there was a negative relationship between the abundances of mites and *Lankesterella* spp. (Table 4). Furthermore, the correlation with SVL was positive in both host species, but this was stronger in *A. erythrurus* (Fig. 4). Both sexes of the two host species presented an abundance peak of *Lankesterella* spp. in August, which was higher in females. Females presented higher abundances of *Lankesterella* spp. overall (Fig. 1).

The interaction between habitat type and species significantly explained the abundance of *Schellackia* spp. (Table 4). The infection abundance of *Schellackia* spp. in *A. erythrurus* was low in both habitats (Fig. 3). In *P. algirus*, the abundance of *Schellackia* spp. in the forest was considerably higher than that of *A. erythrurus* in the same habitat, and also significantly higher than for *P. algirus* in the dunes (Fig. 3, Table 2).

### Gravidity of females and parasites

Gravidity of females did not have a significant effect on the prevalence of mites and blood parasites of any of the three studied species (Table 5). Abundance of mites and blood parasites was not affected by gravidity in the case of *A. erythrurus*, while gravid females of *P. algirus* and *P. edwardsianus* showed higher abundances of mites than non-gravid females of the same species (Table 5).

**Table 5.** Robust estimates and general linear models showing the effect of host gravidity status on parasite abundance and prevalence: estimate (*E*_st_), standard error (s.e.), residual deviance (Dev), residual degrees of freedom (d.f.) and *z*, *F* and *P* statistics

		Abundance			
Host	Parasite	*E*_st_	s.e.	z	*P*
*A. erythrurus*	Mites	0.532	0.280	1.895	0.058
	*Lankesterella* spp.	0.688	0.440	1.564	0.118
	*Schellackia* spp.	1.020	1.324	0.771	0.441
*P. algirus*	Mites	**0.870**	**0.398**	**2.186**	**0.028**
	*Lankesterella* spp.	–	–	–	–
	*Schellackia* spp.	0.693	0.642	1.079	0.281
*P. edwardsianus*	Mites	**2.066**	**0.396**	**5.209**	**<0.001**
	*Lankesterella* spp.	–	–	–	–
	*Schellackia* spp.	–	–	–	–

		Prevalence			
Host	Parasite	Dev	d.f.	*F*	*P*
*A. erythrurus*	Mites	1.528	52	1.529	0.216
	*Lankesterella* spp.	0.507	52	0.507	0.476
	*Schellackia* spp.	0.746	52	0.747	0.388
*P. algirus*	Mites	2.057	28	2.058	0.151
	*Lankesterella* spp.	1.162	28	1.162	0.281
	*Schellackia* spp.	0.196	28	0.196	0.658
*P. edwardsianus*	Mites	0.724	13	0.724	0.395
	*Lankesterella* spp.	–	–	–	–
	*Schellackia* spp.	–	–	–	–

Significant results (*P* > 0.05) are highlighted in bold.

### Climatic variables and parasites

Environmental temperature, but not precipitation, positively correlated with the infection parameters analysed (Tables S3 and S4). This relationship was generally stronger in females than in males (Tables S3 and S4). In the males of the three host lizards, the mean and median infection intensity of *Schellackia* spp. (i.e. considering only infected individuals *sensu* Rózsa *et al*., 2000) were positively correlated with all the temperature parameters calculated. In females of *A. erythrurus* and *P. algirus*, the mean environmental temperature was positively correlated with the prevalence and the mean intensities of *Lankesterella* spp. and *Schellackia* spp., respectively. In females of *P. algirus*, the mean maximum temperature was positively correlated with the prevalence and intensity of *Schellackia* spp. (Table S4). In addition, the mean minimum temperature was positively correlated with the prevalence of *Schellackia* spp. in *P. algirus*.

## Discussion

The results show that coexisting lizard species neither share the same parasites nor a common host–parasite dynamics pattern. In the case of blood parasites, the species *P. edwardsianus* showed a nearly null affection. The genus *Lankesterella* spp. infected almost exclusively *A. erythrurus*, while the genus *Schellackia* spp. infected the three lizard species studied. *Lankesterella* spp. was previously reported infecting *A. erythrurus* (Megía-Palma *et al*., 2014), and consistently with our results, it rarely infects other lizards in the Iberian Peninsula (Maia *et al*., 2014).

The almost null prevalence of blood parasites found in *P. edwardsianus* provided only partial support for the hypothesis that connects age with time of exposure to infection (Maia *et al*., 2014). Nonetheless, the presence of *Schellackia* spp. in *P. edwardsianus* represents the first infection record for lizards of this species. The more frequent infection of *A. erythrurus*, which has an intermediate life span as compared to the two species of *Psammodromus*, supports host–parasite compatibility as the stronger explanation (or partial explanation) for infection patterns in this lizard community (e.g. Megía-Palma *et al*., 2018*a*).

Body size and age are closely related traits in lizards (e.g. Olsson and Shine, 1996). However, Watkins and Blouin-Demers (2019) found that body size, but not age indirectly estimated by skeletochronology, predicted mite load in *Sceloporus clarkii*. In our study, *A. erythrurus* was infected by blood parasites only in individuals with an estimated age older than 300 days. Interestingly, 300 days is the age when the lizards reach sexual maturity (Drechsler and Monrós, 2019). This suggests that the likelihood of acquiring this infection increases at maturation. A potential explanation is that, at maturity, the energy budget initially allocated to immunity is reallocated to reproduction (e.g. French *et al*., 2007; Huyghe *et al*., 2010). However, hormonal levels alone, also associated with sexual maturity, may not be determinant of blood parasite infection because sex, and gravidity status, had no significant effects on the prevalence or the abundance of this parasite. This result contrasts with New World *Lankesterella occidentalis*, which was almost exclusively infecting males of *Sceloporus occidentalis* (Megía-Palma *et al*., 2018*b*). Body length was a significant predictor of mites and *Lankesterella* spp., while body condition was negatively correlated with the abundance of *Lankesterella* spp., indicating that longer but thinner lizards are often infected by this parasite. However, without an experimental approach, we cannot distinguish between potential negative effects of *Lankesterella* spp. on the body condition of *A. erythrurus*, or that weaker lizards were more susceptible to the infection.

Mites are the potential transmitters of *Lankesterella* spp. (e.g. Lainson, 1960), but the fact that mites were found infesting lizards at younger states of the host (the age of youngest infested lizard was estimated in 22 days) than blood parasites, suggests that vectors other than mites might transmit *Lankesterella* spp. to lizards in the studied area. Supporting this hypothesis, lizards captured in the forest held mites and *Lankesterella* spp. opposing abundances in the case of *P. algirus*, although the abundances of mites and *Lankesterella* spp. were both higher in this habitat for *A. erythrurus*. Thus, potential vectors of *Lankesterella* spp., such as sand flies (Diptera: Psychodidae; Telford, 2009), could find more heterogeneity of available microhabitats to thrive in the forest (Neal *et al*., 2016; Megía-Palma *et al*., 2017). Beside sand flies, other haematophagous dipterans feeding on lizards exist in the Mediterranean region, and thus, are potential vectors of haemococcidians. A previous meta-barcoding analysed the presence of reptile DNA in blood meals of mosquitoes and found *Culex hortensis* and *Culex pipiens* (Diptera: Culicidae) feeding on the lizards *Podarcis muralis* and *Lacerta* sp. (Martínez-de la Puente *et al*., 2015). *Culex pipiens* more commonly feeds on humans and other mammals, and is widely present in the Albufera de Valencia; *C. hortensis* is more specialized in reptiles (Martínez-de la Puente *et al*., 2015). An analysis of their vectorial competence to transmit blood parasites to lizards would be illuminating.

The absence of haemogregarines (e.g. genera *Hepatozoon* or *Karyolysus*) in the studied area despite these being common parasites of lizards (Haklová-Kočíková *et al*., 2014; Megía-Palma *et al*., 2020*a*, 2020*b*), including *P. algirus* (Álvarez-Ruiz *et al*., 2018) suggests that environmental conditions in the Albufera de Valencia favour the transmission of haemococcidians, but, for some reason, not other common parasites of lizards. This highlights questions on vectorial competence as well as vector diversity (O'Donoghue, 2017). For example, the significant difference in seasonal variation in mite abundance across lizard species suggested that the susceptibility to the infestation by mites is host-specific. In this sense, the spatial segregation of the three lizard species in this ecosystem might influence their susceptibility to the acquisition of questing mites (e.g. Toyama *et al*., 2019). Our results are consistent with previous studies in bird communities, where difference in life history traits of hosts rather than nest composition (i.e. environment) was proposed as an explanation to the observed differences in the abundance of haematophagous mites between host species (Moreno *et al*., 2009; Cantarero *et al*., 2013; Arce *et al*., 2018). However, at this stage, we cannot rule out that the observed significant difference in mite phenology on the different hosts investigated could also reflect that different species of mites infest different host species in this lizard community. We recommend future research in the Albufera de Valencia directed to identify the haematophagous mites on the lizards and, eventually, the description of likely new mite taxa that increased the biodiversity value of this singular ecosystem.

The forest habitat, where *Lankesterella* spp. was more abundant, presented higher abundances of lizards as well, especially *P. algirus*. This might be explained by an increased transmission favoured by host density (e.g. Lloyd-Smith *et al*., 2005) and/or a higher degree of intra- and interspecific social interactions increasing stress levels in lizards, which might negatively affect their anti-parasitic defences (May and Anderson, 1979; Oppliger *et al*., 1998; Downes and Bauwens, 2002). The negative effects of crowded populations on lizards can be contingent on the habitat quality and resource availability (Oppliger *et al*., 1998). In this sense, the higher proportion of individuals with broken or regenerated tails (Table S4) suggested a higher competition in the forest, where lizards were more abundant (Itescu *et al*., 2017). Our data suggest that haemococcidians, similarly to haemogregarines, can be also biomarkers of competitive stress in lizards because *Schellackia* spp. was more abundant in *P. algirus* captured in the habitat with the higher abundance of lizards and these had a higher proportion of broken tails (Table S4) (Oppliger *et al*., 1998; Lazić *et al*., 2017; Megía-Palma *et al*., 2020*a*). However, this result needs to be taken cautiously, as an increased proportion of broken tails could also mirror a higher predator abundance, which is another source of stress. Further research to clarify this point is needed.

Previous studies reported higher abundances of mites in male *P. algirus* consistently along an environmental gradient (Álvarez-Ruiz *et al*., 2018). Higher levels of steroids in males may provoke immunosuppression and increased susceptibility to parasites (Folstad and Karter, 1992; Belliure *et al*., 2004). However, this expectation does not conform to our results because females in the two species of the genus *Psammodromus* had more mites during the summer, and this effect was stronger in gravid females. Sex-reverse patterns of parasite infection in lizards have been associated before with stressful environments (Megía-Palma *et al*., 2020*a*). Energy allocated to anti-parasitic defences can be reallocated to egg production in gravid females, suggesting that higher mite abundances in females during the summer might reflect this trade-off. This effect was less evident in *A. erythrurus*, a species achieving field body temperatures of 38.8°C (Belliure, 2015), compared to the lower 31.4°C of *Psammodromus* spp. (Carretero and Llorente, 1995) and, hence, conforming to the higher thermal tolerance expected for the former species. Despite the remarkable differences in thermal tolerance of both genera of lizards, the abundances of the three species, particularly during the warmest months, were similar. This suggested that none of the three lizard species ceased their activity during the most adverse season (summer) despite the costs imposed by a thermally restrictive environment due to high temperatures.

In support of the thermal sensitivity hypothesis, *P. algirus* also had stronger infestation by mites in the dunes. This habitat, with low vegetation cover and high abundance of mites, likely represents a habitat of poorer thermal quality for *P. algirus*, a species that demonstrates preferences for habitats with more thermal heterogeneity (Carrascal *et al*., 1989). Furthermore, the results show also sexual differences in the relationship between temperature and *Schellackia* spp. abundance and prevalence. In line with the thermal sensitivity hypothesis, the positive relationship between haemococcidian infection and temperature scores supports the idea that the intensity of infection by *Schellackia* spp. can reflect the higher sensitivity of the females of *Psammodromus* to environmental stress associated with the hot temperatures during the summer.

In conclusion, a combination of intrinsic (species, sex, body size) and extrinsic (season, habitat, temperature) factors were important predictors of parasite abundance, intensity and prevalence. Significant predictors were mostly not generalizable. Nonetheless, although with opposing trends in some species, environmental effects of habitat and temperature supported mites and haemococcidians as biomarkers of environmental quality. Remarkably, the lack of haemogregarines in the lizard community of the Albufera de Valencia suggests that ecological particularities of this place may favour the presence of haemococcidians over other blood parasites. Future studies should investigate the diversity of vectors and their competence to transmit haemococcidians for an integral understanding of the host–parasite webs of this ecosystem. A growing body of evidence supports the potential use of parasites of lizards as biomarkers of environmental perturbation.

## Supplementary material

For supplementary material accompanying this paper visit https://doi.org/10.1017/S0031182021000858. click here to view supplementary material
