# Supplementary material for: Phenological and intrinsic predictors of mite and haemacoccidian infection dynamics in a Mediterranean community of lizards
Source: Parasitology. 2021 Jun 3;148(11):1328–38. doi: 10.1017/S0031182021000858 (PMC8383277; doi:10.1017/S0031182021000858)
Supplement: Supplementary file 1 [file S0031182021000858sup.zip › S0031182021000858sup001.docx]

**Supplementary Material for *Phenological and intrinsic predictors of mite and haemacoccidian infection dynamics in a Mediterranean community of lizards***

**Table S1:** Mean ± SE of lizard abundance of each species in both considered habitat types, with the corresponding sample size (n, in sampling days) and the results of the statistical analysis in each case. Significant results (*P* < 0.05) are highlighted in bold.

| **Abundance (lizards/hour)** | | | | | |
| --- | --- | --- | --- | --- | --- |
|  | **Habitat** | | **ANOVA** | | |
| **Species** | **Forest** | **Dunes** | **df \| n** | **F** | ***P*** |
| *A. erythrurus* | 7.1 ± 0.4 (n = 51) | 5.8 ± 0.4 (n = 53) | 1 \| 104 | 5.497 | **0.021** |
| *P. algirus* | 2.3 ± 0.3 (n = 51) | 1.5 ± 0.1 (n = 53) | 1 \| 104 | 7.868 | **0.006** |
| *P. edwardsianus* | 6.1 ± 1.5 (n = 51) | 1.8 ± 1.2 (n = 53) | 1 \| 104 | 130.2 | **< 0.001** |

**Table S2:** Robust estimate of the variation of parasite abundances with the age of *A. erythrurus*. Significant results (*P* > 0.05) are highlighted in bold.

| **Parasite** | **z** | ***P*** |
| --- | --- | --- |
| Mites | 3.392 | **< 0.001** |
| *Lankesterella* | 24.730 | **< 0.001** |
| *Schellackia* | 0.272 | 0.785 |

**Table S3:** Results for males of the correlation between the monthly prevalence, mean, and median intensity of each parasite with mean temperature, mean maximum temperatures, mean minimum temperatures and accumulated precipitation (mm) for the total dataset and each host species separately. Significant (*P* < 0.05) results are highlighted in bold.

|  | **Mean T (ºC)** | | **Mean Tmax (ºC)** | | **Mean Tmin (ºC)** | | **Prec (mm)** | |
| --- | --- | --- | --- | --- | --- | --- | --- | --- |
| **Prevalence** | **rho** | ***P*** | **rho** | ***P*** | **rho** | ***P*** | **rho** | ***P*** |
| Mites Total | 0.406 | 0.425 | 0.250 | 0.633 | 0.203 | 0.700 | -0.348 | 0.499 |
| Mites A. e. | 0.371 | 0.497 | 0.319 | 0.538 | 0.257 | 0.658 | -0.200 | 0.714 |
| Mites P. a. | -0.247 | 0.637 | -0.172 | 0.744 | -0.247 | 0.637 | 0.185 | 0.725 |
| Mites P. e. | 0.600 | 0.350 | 0.667 | 0.219 | 0.600 | 0.350 | -0.300 | 0.683 |
| *Lankesterella* Total | -0.086 | 0.919 | -0.319 | 0.538 | -0.257 | 0.658 | 0.143 | 0.803 |
| *Lankesterella* A. e. | 0.029 | 1.000 | -0.232 | 0.658 | -0.143 | 0.803 | 0.086 | 0.919 |
| *Lankesterella* P. a. | 0.131 | 0.805 | 0.133 | 0.802 | 0.131 | 0.805 | 0.393 | 0.441 |
| *Schellackia* Total | 0.429 | 0.419 | 0.406 | 0.425 | 0.371 | 0.497 | -0.600 | 0.242 |
| *Schellackia* A. e. | -0.152 | 0.774 | 0.154 | 0.771 | 0.030 | 0.955 | -0.213 | 0.686 |
| *Schellackia* P. a. | 0.278 | 0.594 | -0.047 | 0.930 | 0.062 | 0.908 | -0.031 | 0.954 |

**Continuation of Table S3.**

|  | **Mean T (ºC)** | | **Mean Tmax (ºC)** | | **Mean Tmin (ºC)** | | **Prec (mm)** | |
| --- | --- | --- | --- | --- | --- | --- | --- | --- |
| **Mean intensity** | **rho** | ***P*** | **rho** | ***P*** | **rho** | ***P*** | **rho** | ***P*** |
| Mites Total | -0.257 | 0.658 | -0.406 | 0.425 | -0.486 | 0.356 | -0.429 | 0.419 |
| Mites A. e. | 0.029 | 1.000 | -0.116 | 0.827 | -0.143 | 0.803 | -0.314 | 0.564 |
| Mites P. a. | 0.131 | 0.805 | 0.133 | 0.802 | 0.131 | 0.805 | 0.393 | 0.441 |
| Mites P. e. | -0.359 | 0.553 | -0.237 | 0.701 | -0.359 | 0.553 | 0.308 | 0.614 |
| *Lankesterella* Total | 0.143 | 0.803 | -0.116 | 0.827 | 0.029 | 1.000 | 0.314 | 0.564 |
| *Lankesterella* A. e. | 0.143 | 0.803 | -0.116 | 0.827 | 0.029 | 1.000 | 0.314 | 0.564 |
| *Lankesterella* P. a. | 0.131 | 0.805 | 0.133 | 0.802 | 0.131 | 0.805 | 0.393 | 0.441 |
| *Schellackia* Total | 0.943 | **0.017** | 0.870 | **0.024** | 0.886 | **0.033** | -0.429 | 0.419 |
| *Schellackia* A. e. | -0.152 | 0.774 | 0.062 | 0.908 | -0.030 | 0.955 | -0.273 | 0.600 |
| *Schellackia* P. a. | 0.516 | 0.295 | 0.277 | 0.595 | 0.395 | 0.439 | 0.213 | 0.686 |
| **Median intensity** |  |  |  |  |  |  |  |  |
| Mites Total | -0.232 | 0.658 | -0.368 | 0.473 | 0.464 | 0.354 | -0.551 | 0.257 |
| Mites A. e. | 0.058 | 0.913 | -0.074 | 0.890 | -0.145 | 0.784 | -0.754 | 0.084 |
| Mites P. a. | 0.131 | 0.805 | 0.133 | 0.802 | 0.131 | 0.805 | 0.393 | 0.441 |
| Mites P. e. | -0.359 | 0.553 | -0.237 | 0.701 | -0.359 | 0.553 | 0.308 | 0.614 |
| *Lankesterella* Total | -0.029 | 1.000 | -0.232 | 0.658 | -0.086 | 0.919 | 0.371 | 0.497 |
| *Lankesterella* A. e. | -0.029 | 1.000 | -0.232 | 0.658 | -0.086 | 0.919 | 0.371 | 0.497 |
| *Lankesterella* P. a. | 0.131 | 0.805 | 0.133 | 0.802 | 0.131 | 0.805 | 0.393 | 0.441 |
| *Schellackia* Total | 0.943 | **0.017** | 0.870 | **0.024** | 0.886 | **0.033** | -0.429 | 0.419 |
| *Schellackia* A. e. | -0.152 | 0.774 | 0.062 | 0.908 | -0.030 | 0.955 | -0.273 | 0.600 |
| *Schellackia* P. a. | 0.516 | 0.295 | 0.277 | 0.595 | 0.395 | 0.439 | 0.213 | 0.686 |

**Table S4:** Results for females of the correlation between the monthly prevalence, mean, and median intensity of each parasite with mean temperature, mean maximum temperatures, mean minimum temperatures and accumulated precipitation (mm) for the total dataset and each host species separately. Significant (*P* < 0.05) results are highlighted in bold.

|  | **Mean T (ºC)** | | **Mean Tmax (ºC)** | | **Mean Tmin (ºC)** | | **Prec (mm)** | |
| --- | --- | --- | --- | --- | --- | --- | --- | --- |
| **Prevalence** | **rho** | ***P*** | **rho** | ***P*** | **rho** | ***P*** | **rho** | ***P*** |
| Mites Total | 0.200 | 0.714 | -0.058 | 0.913 | -0.086 | 0.919 | -0.486 | 0.356 |
| Mites A. e. | 0.174 | 0.742 | -0.044 | 0.934 | -0.116 | 0.827 | -0.754 | 0.084 |
| Mites P. a. | 0.116 | 0.827 | 0.044 | 0.934 | -0.087 | 0.870 | -0.783 | 0.066 |
| Mites P. e. | -0.203 | 0.700 | -0.426 | 0.399 | 0.319 | 0.538 | 0.203 | 0.700 |
| *Lankesterella* Total | 0.771 | 0.103 | 0.638 | 0.173 | 0.714 | 0.136 | -0.086 | 0.919 |
| *Lankesterella* A. e. | 0.817 | **0.050** | 0.691 | 0.128 | 0.754 | 0.084 | -0.203 | 0.700 |
| *Lankesterella* P. a. | 0.131 | 0.805 | 0.133 | 0.802 | 0.131 | 0.805 | 0.393 | 0.441 |
| *Schellackia* Total | 0.880 | **0.021** | 0.924 | **0.008** | 0.880 | **0.021** | -0.333 | 0.518 |
| *Schellackia* A. e. | 0.439 | 0.383 | 0.566 | 0.242 | 0.439 | 0.383 | -0.338 | 0.512 |
| *Schellackia* P. a. | 0.941 | **0.005** | 0.924 | **0.008** | 0.941 | **0.005** | -0.152 | 0.774 |

**Continuation of Table S4.**

|  | **Mean T (ºC)** | | **Mean Tmax (ºC)** | | **Mean Tmin (ºC)** | | **Prec (mm)** | |
| --- | --- | --- | --- | --- | --- | --- | --- | --- |
| **Mean intensity** | **rho** | ***P*** | **rho** | ***P*** | **rho** | ***P*** | **rho** | ***P*** |
| Mites A. e. | -0.058 | 0.913 | -0.044 | 0.934 | -0.174 | 0.742 | -0.522 | 0.288 |
| Mites P. a. | -0.116 | 0.827 | -0.309 | 0.552 | -0.377 | 0.462 | -0.493 | 0.321 |
| Mites P. e. | -0.435 | 0.389 | -0.603 | 0.205 | -0.551 | 0.257 | -0.319 | 0.538 |
| *Lankesterella* Total | 0.886 | **0.033** | 0.725 | 0.103 | 0.771 | 0.103 | -0.143 | 0.803 |
| *Lankesterella* A. e. | 0.886 | **0.033** | 0.725 | 0.103 | 0.771 | 0.103 | -0.143 | 0.803 |
| *Lankesterella* P. a. | 0.131 | 0.805 | 0.133 | 0.802 | 0.131 | 0.805 | 0.393 | 0.441 |
| *Schellackia* Total | 0.759 | 0.080 | 0.832 | **0.040** | 0.759 | 0.080 | -0.273 | 0.600 |
| *Schellackia* A. e. | 0.372 | 0.468 | 0.463 | 0.355 | 0.372 | 0.468 | -0.068 | 0.899 |
| *Schellackia* P. a. | 0.759 | 0.080 | 0.832 | **0.040** | 0.759 | 0.080 | -0.273 | 0.600 |
| **Median intensity** |  |  |  |  |  |  |  |  |
| Mites Total | -0.696 | 0.125 | -0.721 | 0.106 | -0.754 | 0.084 | 0.116 | 0.827 |
| Mites A. e. | -0.736 | 0.096 | -0.627 | 0.183 | -0.677 | 0.140 | 0.235 | 0.653 |
| Mites P. a. | -0.088 | 0.868 | -0.269 | 0.607 | -0.353 | 0.492 | -0.618 | 0.191 |
| Mites P. e. | -0.435 | 0.389 | -0.603 | 0.205 | -0.551 | 0.257 | 0.319 | 0.538 |
| *Lankesterella* Total | 0.600 | 0.242 | 0.377 | 0.461 | 0.429 | 0.419 | -0.029 | 1.000 |
| *Lankesterella* A. e. | 0.600 | 0.242 | 0.377 | 0.461 | 0.429 | 0.419 | -0.029 | 1.000 |
| *Lankesterella* P. a. | 0.131 | 0.805 | 0.133 | 0.802 | 0.131 | 0.805 | 0.393 | 0.441 |
| *Schellackia* Total | 0.759 | 0.080 | 0.831 | **0.040** | 0.759 | 0.080 | -0.273 | 0.600 |
| *Schellackia* A. e. | 0.372 | 0.468 | 0.377 | 0.461 | 0.372 | 0.468 | -0.068 | 0.899 |
| *Schellackia* P. a. | 0.759 | 0.080 | 0.831 | **0.040** | 0.759 | 0.080 | -0.273 | 0.600 |

**Table S5:** Proportions of individuals with broken or regenerated tails of each species in both considered habitat types, with the corresponding sample size (n). Additionally, the results of the statistical analysis in each case. Significant results (*P* < 0.05) are highlighted in bold.

| **Proportion of broken tails (%)** | | | | |
| --- | --- | --- | --- | --- |
|  | **Habitat** | | **Chi - square** | |
| **Species** | **Forest** | **Dunes** | **X²** | ***P*** |
| *A. erythrurus* | 30.4 (n = 168) | 25.3 (n = 281) | 1.380 | 0.241 |
| *P. algirus* | 43.9 (n = 66) | 25.0 (n = 100) | 6.500 | **0.012** |
| *P. edwardsianus* | 19.8 (n = 106) | 13.8 (n = 80) | 1.180 | 0.278 |


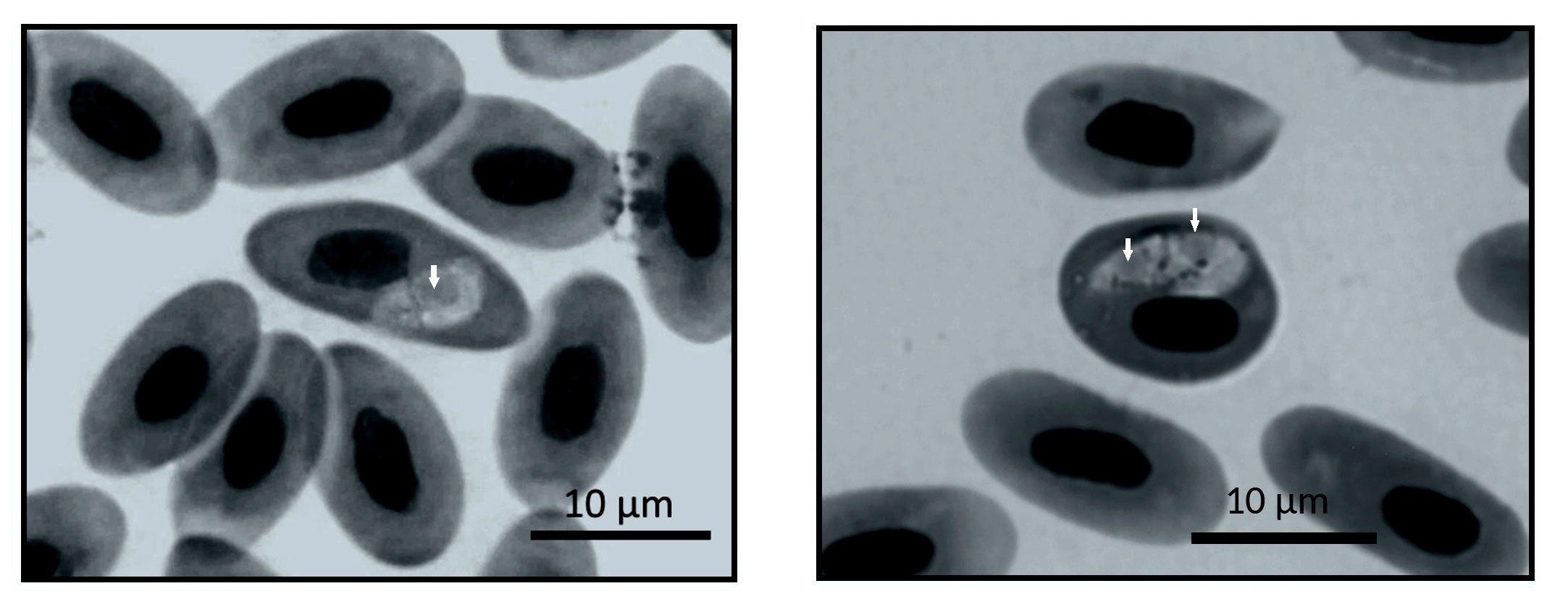
**Figure S1***:* Microscopic photographs of *Schellackia* spp., with one refractile body (left), and *Lankesterella* spp., with two refractile bodies (right). Refractile bodies are indicated with arrows.

**Figure S2:** Monthly variation of mean ± SE host lizard abundance*: A. erythrurus* (solid line)*, P. algirus* (dotted line) and
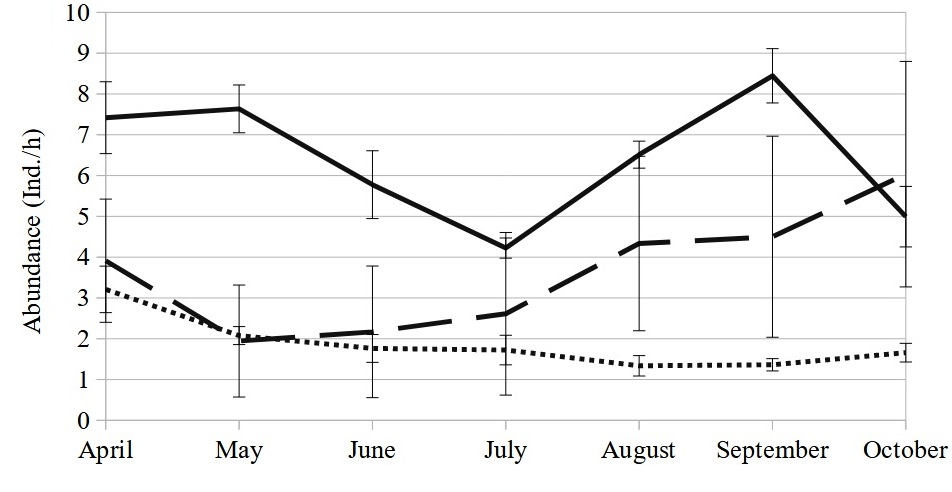
*P. edwardsianus* (dashed line).
