# Supplementary figures and images for: Phenological and intrinsic predictors of mite and haemacoccidian infection dynamics in a Mediterranean community of lizards
Source: Parasitology. 2021 Jun 3;148(11):1328–38. doi: 10.1017/S0031182021000858 (PMC8383277; doi:10.1017/S0031182021000858)

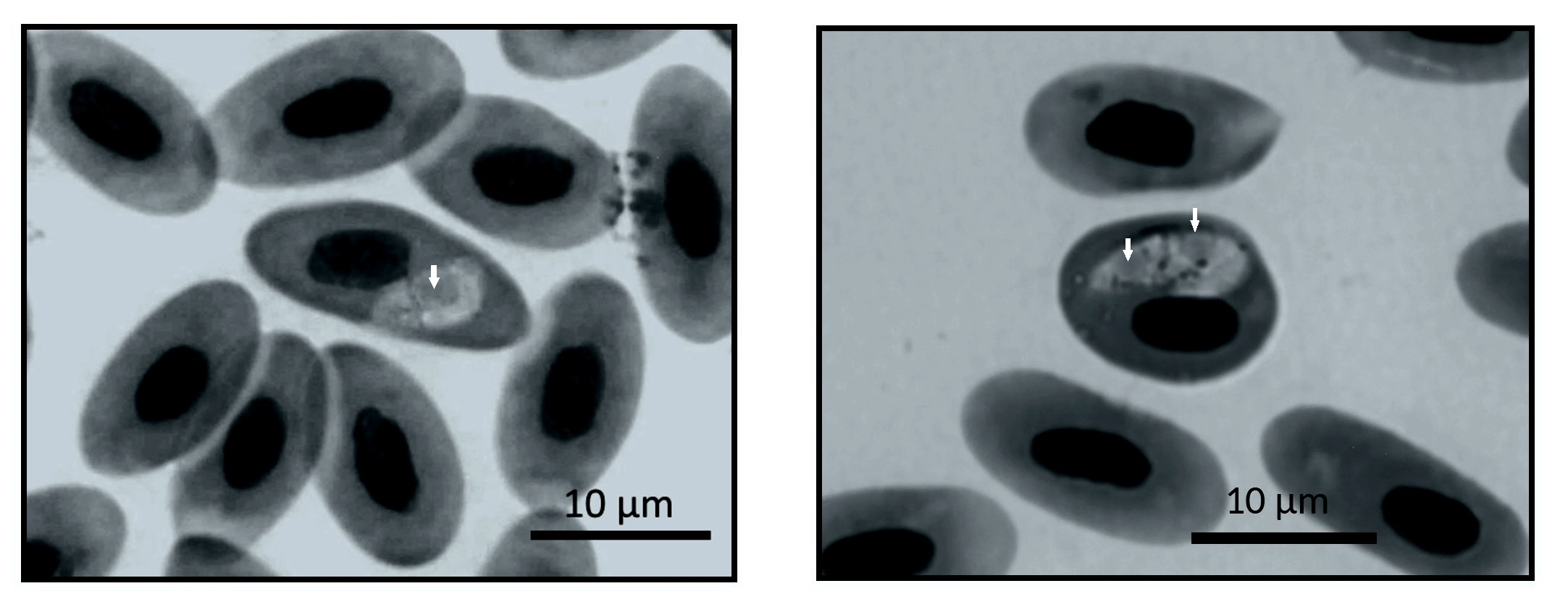

Supplement: Supplementary file 1 [file S0031182021000858sup.zip › S0031182021000858sup002.tif]

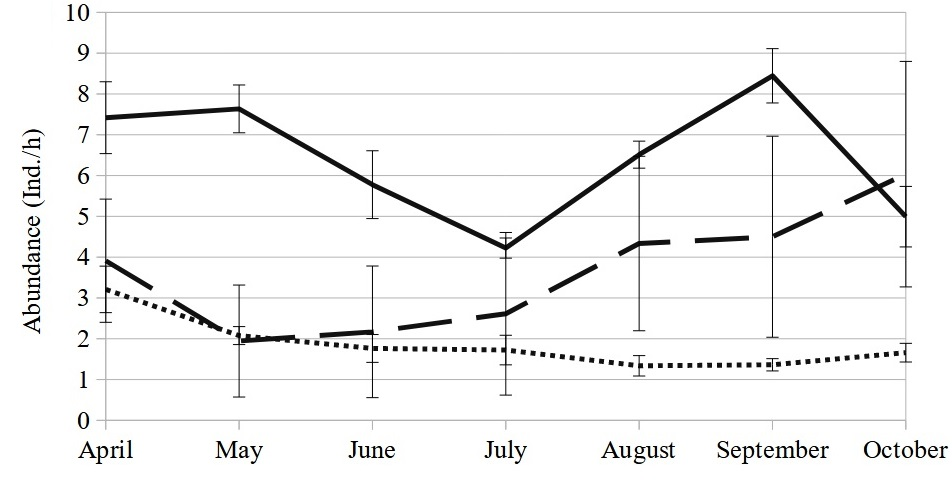

Supplement: Supplementary file 1 [file S0031182021000858sup.zip › S0031182021000858sup003.tif]

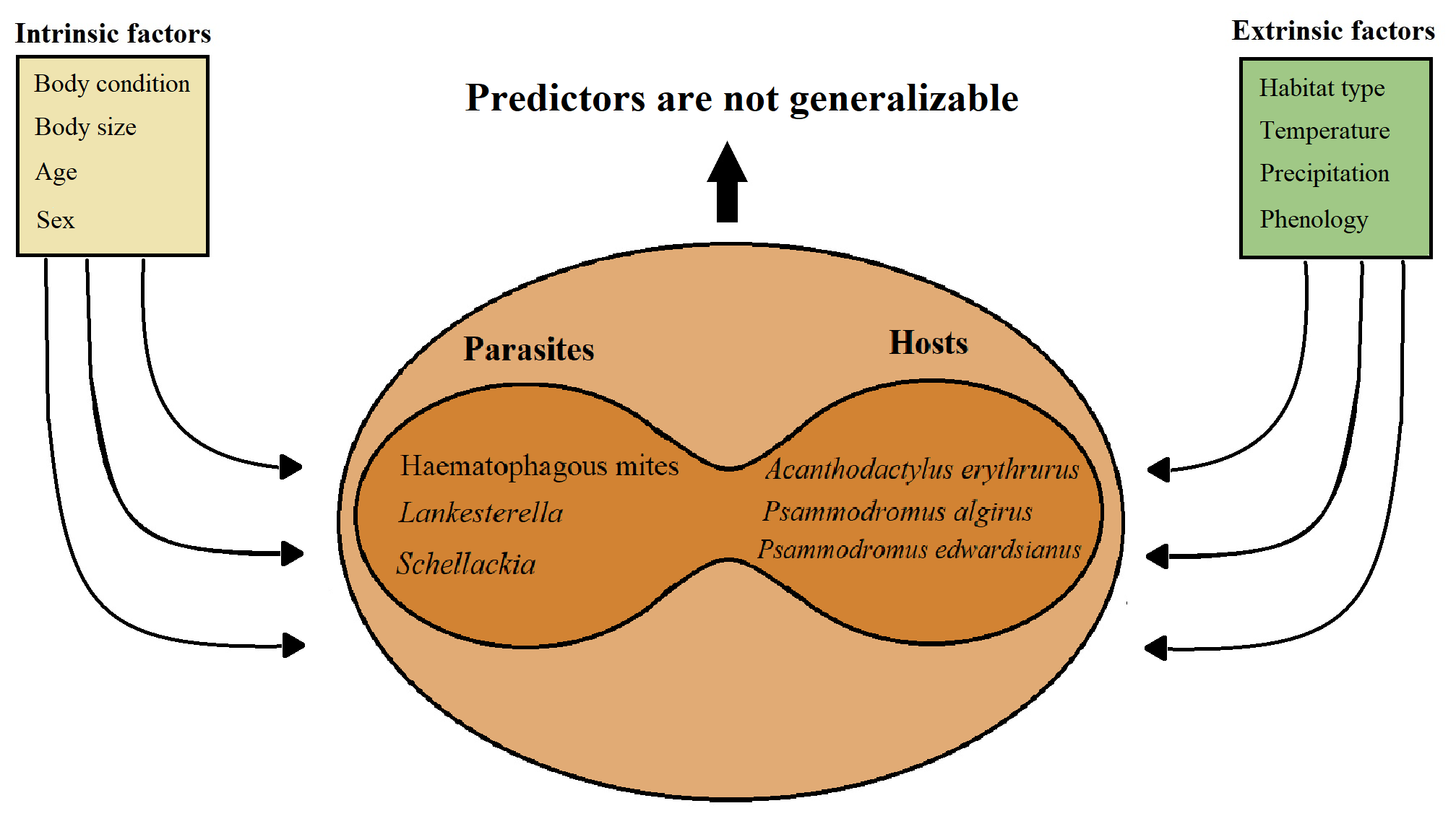

Supplement: Supplementary file 1 [file S0031182021000858sup.zip › S0031182021000858sup004.tif]
